# Supplementary material for: Some like it hot: population-specific adaptations in venom production to abiotic stressors in a widely distributed cnidarian
Source: BMC Biol. 2020 Sep 9;18:121. doi: 10.1186/s12915-020-00855-8 (PMC7488265; doi:10.1186/s12915-020-00855-8)

Figure S1. Change in oxygen consumption following the fishing line treatment (black) and in the untreated control (red).

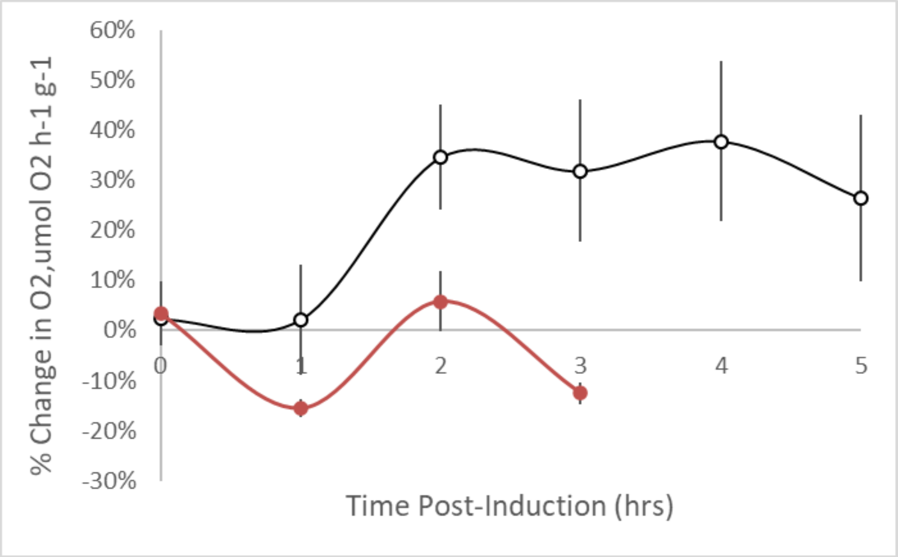

Figure S2. New venom components

S2A. NEP8-like is expressed in nematocytes in larvae and primary polyps and shares high sequence similarity with NEP8 toxin found in nematocysts. Signal peptide is shown in bold (identified by SignalP online tool, http://www.cbs.dtu.dk/services/SignalP/), ShKT domains are highlighted, and cystein residues forming the ShKT motives are in green.

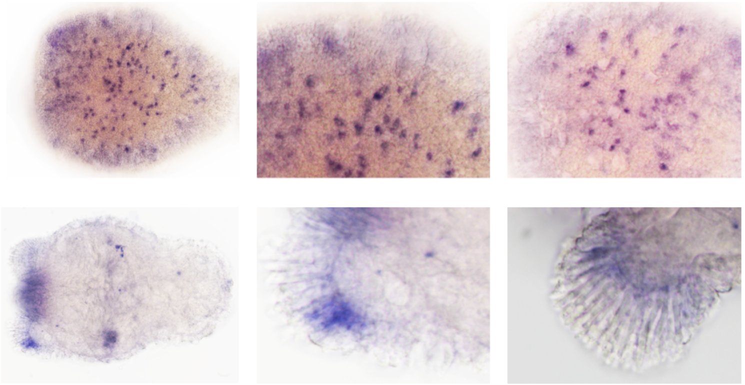

```
Nep8_Nve15921          MLRRPLLLVLFTVFSTLYAKDLRGVSPPTNESEAEVSPGDDEGPPEPGNEPDVNWRTLTI 60
Nep8_like_Nve6865      MASLFWLLVACLVLLAVDAKEIRRKKLYRQ--------------HESLRTIAETLERVHI 46
                       *     ***   *: :: **::*  .   :               *  .    . . : *

Nep8_Nve15921          DPEDCKDKGKDCESLADEGNCLKKLNYAVGNCPWTCRFCKKEN-GDSKKCKDLAGERHCN 119
Nep8_like_Nve6865      SPEDCKDYKSNCEEFADEGSCLKRFDYGFKFCPWSCRFCAKPEKYNTERCADLAGK-RCG 105
                       .******  .:**.:****.***:::*..  ***:**** * :  ::::* ****: :*.

Nep8_Nve15921          GWKVKGDCVRLPDYMMQNCKLSCELCGPETRFKYTDEDVRCPEWAQAGYCSTNTDINLKC 179
Nep8_like_Nve6865      PWAQRSECLRLPEFMQQNCKQSCDLCGPENA--YTDDDVRCPYWGKEGYCRTDAEINSRC 163
                        *  :.:*:***::* **** **:*****.   ***:***** *.: *** *:::** :*

Nep8_Nve15921          PHSCRKYKQRAPASTPYPYPVEALHPYQYRVVLPSVTILQTTTA-----APSTQPAETT- 233
Nep8_like_Nve6865      PYSCRKYRSVSPAPQPYPYPIGTLYPYQPTPVP-GVTVSQPKIVLQPYPAPYPYPYPVPS 222
                       *:*****:. :**  *****: :*:***   *  .**: * . .     **   *  .

Nep8_Nve15921          KAPPNTAAPTAAP------------TPAPTPAPAPAPTPAPAPAPTPAPAPAPATTPAPA 281
Nep8_like_Nve6865      PAPSPSPSPSPAPSPSPSPSPALSPSPAPSPSPSPSPSPSPSPSPSPSPSPSPSPSPSPS 282
                        **  : :*: **            :***:*:*:*:*:*:*:*:*:*:*:*:*: :*:*:

Nep8_Nve15921          PVP---PAPAPAPAPAPAPP-APPVAPAPQTAPLAGSPPESTPEEQDD--------NSA- 328
Nep8_like_Nve6865      PSPEPSPSPAPSPAPEPSPAPSPASEPSPETTTPAPKDPTQAPVVVETQQPSTQLPAPTQ 342
                       * *   *:***:*** *:*  :*   *:*:*:  * . * .:*   :           :

Nep8_Nve15921          DESTEI----EAGEGGGELCDEKHSSQQ-------------------------------- 352
Nep8_like_Nve6865      PPTEPIDSSGNPGESGNGVWPGQKGQPSAAPVTQPQGSGEEEEVEEGVTTDAPVGSPVPE 402
                         :  *    : **.*. :   ::.. .

Nep8_Nve15921          ------------------------------------------------------------ 352
Nep8_like_Nve6865      ATPGPAATNAPEATQAPEVVTQAPEATSAPEVTAAPTGQATPASNPIGTPPVVFDHRKSH 462

Nep8_Nve15921          ------------------------------------------------------------ 352
Nep8_like_Nve6865      RSRNAKNDAHHHNNNNHHNNNNHHNNPHNNAPSTPPTAPPSPAPVFSKKLPVSGLDKATN 522

Nep8_Nve15921          -----------------  352
Nep8_like_Nve6865      AAKAKRNKHGHRHIISE  539
```

S2B. NveSkT1 is expressed in pharyngeal gland cells in planulae and primary polyps. Signal peptide is in bold (identified by SignalP online tool, http://www.cbs.dtu.dk/services/SignalP/), peptydoglycan binding domain is in blue, metallopeptidase is in grey, and ShKT domain is underligned with cysteins in green (domains were identified by Interpro, https://www.ebi.ac.uk/interpro/). The ShKT domain od NveSkT1 shares noticeable sequence similarity with potassium channel blocker ShK from the sea anemone *Stichodactyla helianthus*.

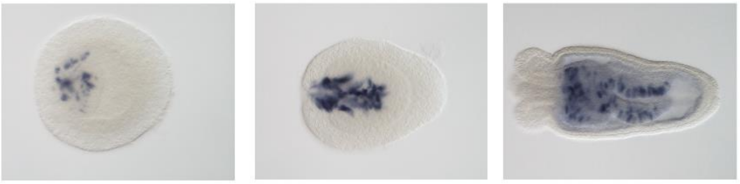

>NveSkT1

MKTLVFAGLLCLIAFAVSEDDDQTMALKYLNQFHYISPARSGNHNVKTALEKFQSFAGLPVTGEIDAATIA
QMKMPRCGMPDVDDDGLRIRRYKLGSKWNKKHLTYHISHGQDLSSSVQDRVFAKALDYWARVSGLTFSRTM
DGENADLKISFGPKSHGGTHDPEGTCSYPFDGPGGVLAHAFFPRNGRAHFDEDEDFTDGTYEGTNLLWVAT
HEFGHSLGLHHSDVRDAVMYPYYTGYKPGFDLKADDIAGIRAHYGDENSGGSQPCVDNNQSCPDWTIYCTN
NEYVIANCRKSCGSC

```
ShK_Stichodactyla         RSCIDTIPKSRCTAFQCKHSMKYRLSFCRKTCGTC   35
NveSkT1-ShKt_domain       -PCVDNNQSCPDWTIYC-TNNEYVIANCRKSCGSC   33
                            *:*.  ..   :: *   . :* :: ***:**:*
```

Figure S3. Gene expression dynamics under UV light stress.

NS – Nova Scotia, ME – Maine, NH – New Hampshire, MA – Massachusetts, NC - North Carolina

S3A) Gene expression dynamics under UV light stress among the populations from the North to the South. If the change in expression of a gene is greater than 2.4 times and $p<0.05$ (Student's t-test), the corresponding data point is outlined in bold.

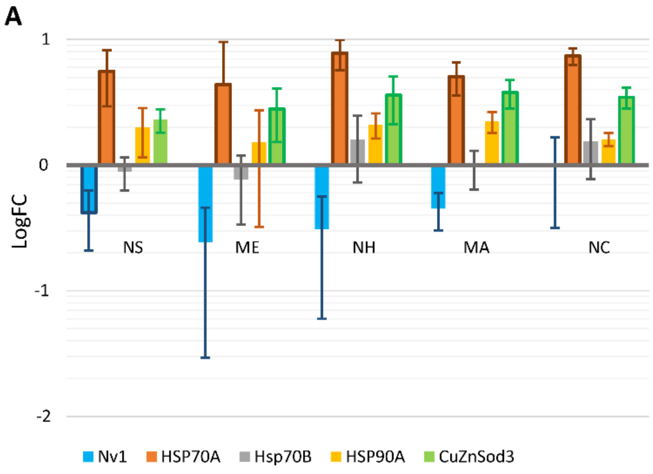

S3B and S3C) Expression of toxins in Massachusetts (B) and North Carolina (C) following UV light stress. Gene expression is represented as Log_{10} of normalized fluorescence units measured by nCounter technology. P-values calculated by Student's t-test are shown for each gene. If the change in expression of a gene is greater than 2.4 times and $p<0.05$, the corresponding p-value is shown in red. MA-ctr-Av – average values for control samples in MA; MA-UV-Av – average values for samples exposed to the UV light in MA; NC-ctr-Av – average values for control samples in NC; NC-UV-Av – average values for samples exposed to the UV light in NC.

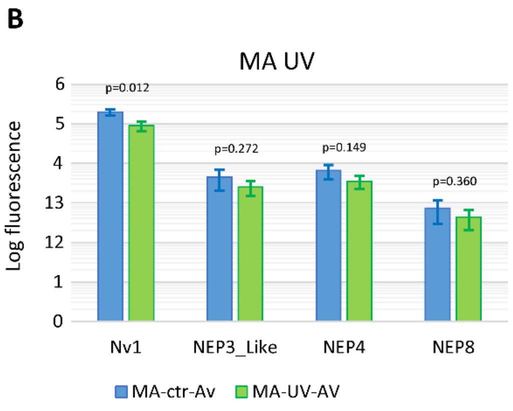

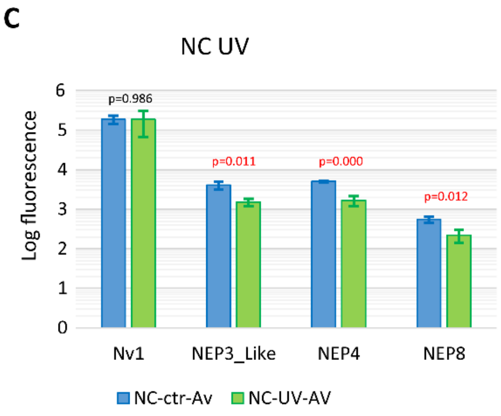

Figure S4. PCA analysis was conducted using the built in Trinity toolkit script PtR.

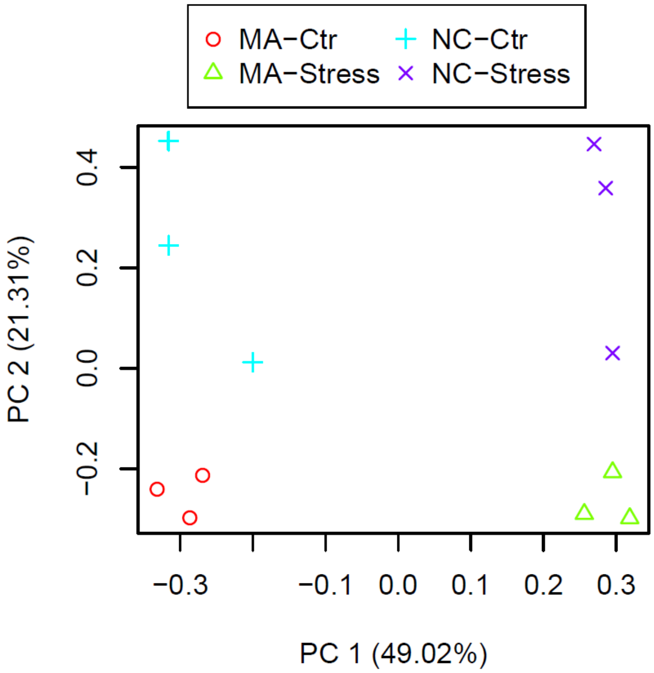

Supplement: Supplementary file 2 — Additional file 2: Fig. S1 – Change in oxygen consumption following the fishing line treatment and in the untreated control. Fig. S2 - New venom components. Fig. S3 - Gene expression dynamics under UV light stress. Fig. S4 - PCA analysis. [file 12915_2020_855_MOESM2_ESM.pdf]
